# The Impact of Type 2 Diabetes on the Efficacy of ADP Receptor Blockers in Patients with Acute ST Elevation Myocardial Infarction: A Pilot Prospective Study

**DOI:** 10.1155/2016/2909436

**Published:** 2016-07-17

**Authors:** Matej Samoš, Marián Fedor, František Kovář, Peter Galajda, Tomáš Bolek, Lucia Stančiaková, Jana Fedorová, Ján Staško, Peter Kubisz, Marián Mokáň

**Affiliations:** ^1^Department of Internal Medicine I, Jessenius Faculty of Medicine in Martin, Comenius University in Bratislava, 03659 Martin, Slovakia; ^2^National Centre of Hemostasis and Thrombosis, Department of Hematology and Blood Transfusion, Jessenius Faculty of Medicine in Martin, Comenius University in Bratislava, 03659 Martin, Slovakia; ^3^HemoMedika, Centre of Thrombosis and Hemostasis, 03601 Martin, Slovakia

## Abstract

*Background*. The aim of this study was to validate the impact of type 2 diabetes (T2D) on the platelet reactivity in patients with acute ST elevation myocardial infarction (STEMI) treated with adenosine diphosphate (ADP) receptor blockers.* Methods*. A pilot prospective study was performed. Totally 67 patients were enrolled. 21 patients had T2D. Among all study population, 33 patients received clopidogrel and 34 patients received prasugrel. The efficacy of ADP receptor blocker therapy had been tested in two time intervals using light transmission aggregometry with specific inducer and vasodilator-stimulated phosphoprotein phosphorylation (VASP-P) flow cytometry assay.* Results*. There were no significant differences in platelet aggregability among T2D and nondiabetic (ND) group. The platelet reactivity index of VASP-P did not differ significantly between T2D and ND group (59.4 ± 30.9% versus 60.0 ± 25.2% and 33.9 ± 25.3% versus 38.6 ± 29.3% in second testing). The number of ADP receptor blocker nonresponders did not differ significantly between T2D and ND patients. The time interval from ADP receptor blocker loading dosing to the blood sampling was similar in T2D and ND patients in both examinations.* Conclusion*. This prospective study did not confirm the higher platelet reactivity and higher prevalence of ADP receptor blocker nonresponders in T2D acute STEMI patients.

## 1. Background

Type 2 diabetes (T2D) is a strong and independent risk factor of cardiovascular disease in both men and women [1–3]. Diabetes not only increases the risk of acute coronary syndrome (ACS) but also increases the mortality associated with this acute event. T2D worsens the course of ACS and increases the risk of its complications including cardiogenic shock and death [4] and remains an independent predictor of worse prognosis of ACS, even after excluding the impact of older age, more severe coronary artery disease, and worse left ventricular function in patients with T2D [5]. Moreover, T2D is connected with platelet dysfunction [6, 7], which increases the risk of arterial thrombosis. Several recently published studies and case reports pointed at a failure in antiplatelet response to clopidogrel (the most frequently used ADP receptor blocker in ACS patients) which might be connected with insulin resistance and T2D. High clopidogrel on-treatment platelet reactivity is associated with increased risk of thrombotic adverse events including stent thrombosis [8–10]. The aim of this preliminary prospective study was to validate the impact of T2D in relation to the on-treatment platelet reactivity in patients with acute ST elevation myocardial infarction (STEMI) treated with ADP receptor blocker therapy.

## 2. Methods

### 2.1. Study Design and Patients

A single centre, pilot prospective observational study in patients with acute STEMI was performed. All patients underwent coronary angiography and primary percutaneous coronary intervention (pPCI) of culprit coronary lesion. Totally 67 consecutive presentations of patients (37 men and 30 women; mean age 67 years; the youngest patient was 34-year-old and the oldest patient was 89-year-old) with acute STEMI and pPCI of coronary lesion were enrolled in this study. Patients with multivessel coronary disease planned for surgical revascularization, patients treated only conservatively, and hemodynamically unstable patients (i.e., patients in Killip class IV) had been excluded from this study. Additionally, patients with hypertensive crisis, kidney, and liver failure had been also excluded from study population. Moreover, patients with medication which could interfere with the action of ADP receptor blockers, such as omeprazole, fluconazole, or morphine were also excluded from this study. All patients should be ADPRB naïve prior to myocardial infarction. Patients with known and correctly diagnosed history of T2D had been assigned to T2D group. This group included 21 patients. In all patients without previous history of T2D the glycated hemoglobin (HbA1C) levels were assessed in order to exclude patients with undiagnosed T2D. Patients with HbA1C levels > 5.7% DCCT were considered to be patients with possibly undiagnosed T2D and these patients had been excluded from the study. Subsequently, a standard oral glucose tolerance test (75 g of glucose was administrated in 100 mL of water and a venous blood sample was taken two hours after the glucose administration) was performed in the rest of the patients without previous history of T2D one month after the hospital discharge. Patients with blood glucose value > 7.8 mmol/L two hours after the glucose administration had been also excluded from the study. Patients without previous history of T2D, with HbA1C levels < 5.7% DCCT and blood glucose value < 7.8 mmol/L shown in oral glucose tolerance test had been assigned to nondiabetic (ND) group. The basic demographic data and concomitant medication in the study population are shown in Table 1. Among all study population, 33 patients received clopidogrel (a loading dose of 600 mg followed by a maintenance dose of 75 mg/daily) and 34 patients received prasugrel (a loading dose of 60 mg followed by a maintenance dose of 10 mg/daily). Totally 22 patients enrolled in this study were >75 years old (9 T2D patients and 13 ND patients); all of these patients were clopidogrel-treated. All patients received aspirin therapy (a loading dose of 200–400 mg followed by a maintenance dose of 100 mg/daily) and a weight-adjusted unfractionated heparin therapy (100 IU/kg intravenously) prior to pPCI in order to prevent periprocedural thrombosis. No other antiplatelet or anticoagulant therapy was administrated in studied population. The drug compliance had been carefully monitored with a healthcare professional, who supervised all antiplatelet drug administration. Venous blood samples had been taken after obtaining the written informed content in all patients enrolled in this study in order to assess the on-treatment platelet reactivity using selected platelet function tests.

### 2.2. Platelet Function Testing

Blood samples had been taken using 3.8% citrate vacutainer blood collection tubes. Blood samples had been immediately analyzed within first 2 hours from blood sampling. The samples had been taken in the following time intervals:

Sample 1, at the time of patient arrival to cath laboratory: this sample aimed to test the efficacy of ADP receptor blocker given in loading doses prior to the urgent coronary angiography and pPCI of coronary lesion.

Sample 2, one hour after the administration of first ADP receptor blocker maintenance dose: this sample aimed to test the efficacy of in-hospital ADP receptor blocker therapy given in maintenance dosage.

The platelet reactivity was tested using light transmission aggregometry (LTA) with specific inducer (ADP) and vasodilator-stimulated phosphoprotein phosphorylation (VASP-P) flow cytometry assay.

*Light Transmission Aggregometry (LTA).* this method represents recently the “golden standard” of platelet function testing. ADP (10 *μ*mol/L) was used as specific inducer for ADP receptor blocker efficacy testing. LTA was examined with Chrono-Log model 700 (Chronolog Corporation, Havertown, PA, USA). The platelet aggregability was assessed on the basis of the change in the plasma turbidity after the addition of the specific inducer. Residual platelet aggregability > 50% after the addition of ADP was considered to be high on-treatment platelet reactivity (HTPR).

*VASP-P Flow Cytometry Assay.* In this analysis, we used PLT VASP/P2Y12 assay kits (Diagnostica Stago, France). Sample of citrate blood was incubated with prostaglandin E1 (PGE1) and PGE1 + ADP (activated platelets). After cellular permeabilization by nonionic detergent, VASP-P is labeled by indirect no-wash immunofluorescence using a specific monoclonal antibody. Dual color flow cytometry analysis then allowed comparison of the 2 tested conditions. Analysis was carried out on FACSCalibur flow cytometer (BD Biosciences, San Jose, California). In the final step, the platelet reactivity index (PRI) was calculated using corrected mean VASP fluorescence intensities (MFIc) in the presence of PGE1 alone (resting platelets) or PGE1 + ADP simultaneously (activated platelets). Index represented the ratio of activated/resting platelets and was calculated according to the following equation:

(1) $$\begin{array}{c} \text{PRI}=\frac{{MFIc}^{PGE1}-{MFIc}^{\left[PGE1+ADP\right]}}{{MFIc}^{PGE1}}\times 100. \end{array}$$

The resulting value described PRI to ADP treatment in the range of 0% to 100%. Values of PRI above 50% were considered as determinant of HTPR and inadequate response to treatment.

### 2.3. Statistical Analysis

Data were in the first step checked for normality with the Shapiro-Wilk test. Normally distributed continuous or interval-scaled variables are presented as mean ± standard deviation (SD); otherwise median (M) and quartile ranges from the lower quartile to the upper quartile were used. Group effects (i.e., differences between T2D and ND groups) were tested with* t*-test in the case of normally distributed data or with Mann-Whitney* U* test when data distribution was asymmetrical. Differences between proportions (e.g., number of patients in T2D and ND groups) were tested with binominal tests. Categorical variables grouped in 2-way contingency tables were analyzed using chi-square tests. The significance of *p* < 0.05 was considered as a criterion for comparison between data sets with equal and unequal variances. The statistical analysis was performed with Statistica v. 7.0 (StatSoft Inc., Dell Software, Tulsa, Oklahoma, USA). Sample size calculation was based on the assumption of the incidence of HTPR among ADP receptor blockers-treated T2D patients reported in previously published studies [9, 10]. The primary aim of this study was to clarify possible differences in ADP receptor blockers on-treatment platelet reactivity according to T2D status. Choosing a two-sided value of 0.05, we estimated that an overall sample size of 20 T2D patients and 20 control (nondiabetic) patients would be sufficient for statistical analysis. To reach even more valid sample, we decided to enroll more than 20 patients in each of the compared groups (however, we were able to enroll only one more T2D patient who met the inclusion criteria of the study). The statistical analysis was consulted with a professional and designed prior to patient enrollment to achieve a valid statistical evaluation of the results of the study.

## 3. Results

The time interval from ADP receptor blocker loading dose administration to the collection of sample 1 was 1.8 ± 0.9 hours and to the collection of sample 2 was 20.5 ± 2.1 hours. The mean platelet aggregability after the induction with ADP was 52.5 ± 23.6% in sample 1 and 39.7 ± 24.5% in sample 2. Examination of VASP phosphorylation showed mean PRI 59.7 ± 26.9% in sample 1 and mean PRI 37.0 ± 27.8% in sample 2, respectively.

When comparing the T2D and ND group (Table 2) there were no significant differences in platelet aggregability after ADP neither in sample 1 nor in sample 2 (sample 1: 57.2 ± 26.4% versus 50.3 ± 22.3%, NS; sample 2: 45.9 ± 31.3% versus 37.0 ± 20.8%, NS). The PRI of VASP-P showed similar results: there were no significant differences between T2D and ND group neither in sample 1 (59.4 ± 30.9% versus 60.0 ± 25.2%, NS) nor in sample 2 (33.9 ± 25.3% versus 38.6 ± 29.3%, NS). The time interval from ADP receptor blocker loading dose administration to the sample collection was similar in T2D and ND patients in both examinations (sample 1: 1.8 ± 0.9 hours versus 1.7 ± 0.9 hours; sample 2: 21.6 ± 2.2 hours versus 20.0 ± 1.9 hours).

Subsequently, an analysis of ADP receptor blocker nonresponders (Table 2) was performed. The difference in the prevalence of ADP receptor blocker nonresponders in T2D and ND patients did not reach statistical significance. However, the number of ADP receptor blocker nonresponders tended to be higher in T2D patients. In T2D patients an incomplete response on ADP receptor blocker (PRI > 50%) was identified in 52.4% of patients in first sample and in 33.3% in second sample. While in ND group, 56.5% of patients were nonresponders in first sample and 30.4% of patients did not respond sufficiently in second sample. No significant differences in the prevalence of ADP blocker nonresponders between T2D prasugrel-treated and ND prasugrel-treated patients were found (sample 1: 40.0% versus 41.7%, NS; sample 2: 10.0% versus 12.5%, NS). In addition, the prevalence of ADP receptor blocker nonresponders did not differ significantly in clopidogrel-treated T2D patients compared to clopidogrel-treated ND patients (sample 1: 63.6% versus 72.7%, NS; sample 2: 54.6 versus 50.0%, NS).

Prasugrel induced in this preliminary study significantly more potent platelet inhibition (Table 3) than that of clopidogrel. Prasugrel-treated patients had significantly lower residual platelet aggregation in both samples: 45.9 ± 26.2% versus 59.2 ± 18.8% (*p* < 0.05) in sample 1 and 24.9 ± 17.7% versus 51.7 ± 22.8% (*p* < 0.001) in sample 2, respectively. Similarly, the PRI of VASP-P was significantly lower in prasugrel-treated patients in sample 1, as well as in sample 2 (sample 1: 52.7 ± 29.7% versus 68.9 ± 19.9, *p* < 0.05; sample 2: 26.3 ± 24.3% versus 51.8 ± 22.8%, *p* < 0.01). Consistently, significantly lower residual platelet reactivity was found in prasugrel-treated T2D patients when compared to clopidogrel-treated T2D patients in both samples (sample 1: 43.3 ± 20.6% versus 63.5 ± 22.8%, *p* < 0.05; sample 2: 22.9 ± 16.9% versus 60.3 ± 26.6%, *p* < 0.01). Significantly better inhibition of ADP signaling pathway in prasugrel-treated T2D patients was demonstrated with significantly lower PRI of VASP-P in both samples (sample 1: 46.8 ± 33.5 versus 78.3 ± 13.0, *p* < 0.05; sample 2: 18.7 ± 15.7 versus 56.8 ± 18.7, *p* < 0.01).

## 4. Discussion

T2D is a strong and independent risk factor of acute STEMI. T2D increases the risk of future complications in acute STEMI patients. Endothelial dysfunction [11], abnormalities in coagulation and fibrinolysis [12], and platelet dysfunction [6, 7] are important factors of worsen prognosis of acute STEMI in T2D patients. However, several studies pointed at a failure in antiplatelet response to clopidogrel which might be connected with insulin resistance and T2D [8–10]. These studies consistently identified higher residual on-treatment platelet reactivity in clopidogrel-treated T2D patients and higher number of clopidogrel nonresponders among diabetic patients. Moreover, HTPR was in these studies consistently connected with higher risk of adverse ischemic events after PCI. The mechanism of this phenomenon remains unclear. The study performed by Erlinge et al. [9] showed that platelets of diabetic patients with clopidogrel HTPR responded well to ex vivo administration of clopidogrel active metabolite. This finding suggests a potential interaction between T2D and pharmacokinetic processes of clopidogrel metabolism.

On the other hand, the results of this preliminary prospective observation did not confirm the significantly higher residual platelet reactivity or significantly higher prevalence of HTPR in T2D acute STEMI patients undergoing pPCI of culprit coronary lesion. The exact explanation of this observation is recently missing. The time interval from drug administration to blood sample collection did not differ significantly in T2D and ND patients neither in sample 1 nor in sample 2. The differences in time interval from drug dosing to blood sampling therefore cannot explain the results obtained in this study.

Another possible explanation may be the fact that the majority of studies pointing on the higher prevalence of HTPR among T2D patients were performed on a sample of stable coronary/ischemic heart disease patients. It is generally accepted that patients with acute STEMI represent a special group with different clinical and risk profile. It is therefore possible that results obtained from the studies on stable patients might not be fully applicable in high risk ACS patients. However, study performed by Cuisset et al. identified high prevalence of clopidogrel nonresponders (50% of patients) also in T2D patients undergoing PCI for ACS [13]. In the light of these data, the real influence of acute coronary syndrome itself on the prevalence of HTPR in T2D patients seems not to be probable.

An alternative explanation of similar on-treatment platelet reactivity in T2D and ND patients in this study might be a possibility that the failure in antiplatelet response is in T2D patients specifically associated with clopidogrel. The administration of newer ADP receptor blockers might not be connected with such a failure in on-treatment response [14]. In fact, prasugrel administration in the TRITON-TIMI 38 trial achieved the most pronounced clinical benefit just in T2D patients [15]. Prasugrel was in our preliminary study quite frequently used among T2D (48% of patients), as well as among ND patients (52% of patients). Prasugrel induced in our study significantly more potent platelet inhibition than that of clopidogrel in both samples. The efficacy of newer ADP receptor blockers among high risk T2D acute coronary syndrome patients was consistently proven in a recent single centre randomized clinical study performed by Laine et al. [16]. It is therefore possible that more frequent use of newer ADP receptor blockers (such as prasugrel) would resolve the insufficient platelet inhibition, which might be seen in clopidogrel-treated T2D patients. Nevertheless, the relationship between T2D and ADP receptor blocker on-treatment platelet reactivity remains inadequately explained [17–19] and further studies would be needed for the final clarification of this issue.

## 5. Limitations

There were some important limitations of our analysis. First, this study had a prospective observational design and not a randomized double blinded one. The decision on ADP receptor blocker therapy strategy (clopidogrel versus prasugrel) was left to the physician who performed the diagnostic ECG record (general practitioner, cardiologist, ED physician, etc.). Therefore the data obtained in this study do not have the evidence power of data from a randomized double blinded trial. Second, a low sample size might be a limitation of the study. A relatively small patient sample cannot guarantee significant power. Third, a relatively short time interval from ADPRB loading dosing to first sampling (especially in the settings of acute coronary syndrome) might be another limitation of this study. It is already known that time to peak platelet inhibition is generally prolonged in acute STEMI patients and also in stable coronary artery disease patients with T2D [19]. However, in this study, we wanted to test the impact of T2D on the on-treatment response in previously ADPRB naïve acute STEMI patients undergoing pPCI. Therefore we decided to test the antiplatelet response prior to pPCI and one day after the procedure rather than after a prolonged ADPRB administration. Nevertheless, this fact could affect the on-treatment platelet reactivity detected in our study. Finally, the drug compliance was not proven by a laboratory testing (measurement of clopidogrel metabolite, etc.). Although, the drug compliance was in this study carefully monitored by a healthcare professional who supervised all antiplatelet drug administrations, the exact confirmation of drug compliance with laboratory assessment is missing. The results of this study should be confirmed in large, similarly designed, randomized trial.

## 6. Conclusion

This prospective study did not confirm the higher residual platelet reactivity and higher prevalence of HTPR in T2D acute STEMI patients undergoing pPCI of culprit coronary lesion. However, the results of this study are preliminary and further studies on larger sample sizes would be definitely needed for the final clarification of this issue.

## Figures and Tables

**Table 1:** Demographic data and concomitant medication in studied acute STEMI patients.

	T2D group	ND group	Significance
Number of patients (men/women)	21 (11/10)	46 (26/20)	NS
T2D duration (years)	11.4 ± 7.6	—	N/A
HbA1C levels	7.2 ± 1.9%	4.03 ± 0.7%	*p* < 0.001
Age	72 (52–89)	65 (34–88)	NS
Body mass index (kg/m^2^)	29.6 ± 4.2	28.6 ± 4.2	NS
Beta blockers (% of patients/number of patients)	90%/19 patients	89%/41 patients	NS
ACE inhibitors or AT1 blockers (% of patients/number of patients)	85%/18 patients	67%/31 patients	NS
Statins (% of patients/number of patients)	100%/21 patients	96%/44 patients	NS
Diuretics (% of patients/number of patients)	52%/11 patients	31%/14 patients	NS
Clopidogrel (% of patients/number of patients)	52%/11 patients	48%/22 patients	NS
Prasugrel (% of patients/number of patients)	48%/10 patients	52%/24 patients	NS

**Table 2:** On-treatment platelet reactivity and prevalence of ADP receptor blocker nonresponders in T2D and ND patients.

On-treatment platelet reactivity	T2D patients	ND patients	Significance
LTA with ADP induction (%)			
Sample 1	57.2 ± 26.4	50.3 ± 22.3	NS
Sample 2	45.9 ± 31.3	37.0 ± 20.8	NS
PRI of VASP phosphorylation (%)			
Sample 1	59.4 ± 30.9	60.0 ± 25.2	NS
Sample 2	33.9 ± 25.3	38.6 ± 29.3	NS

ADP receptor blocker nonresponders (% of patients/number of patients)	T2D patients	ND patients	Significance

ADP receptor blocker nonresponders			
Sample 1	52.4%/11 patients	56.5%/26 patients	NS
Sample 2	33.3%/7 patients	30.4%/14 patients	NS

**Table 3:** Platelet aggregability and PRI of VASP-P in prasugrel- and clopidogrel-treated patients with acute STEMI (analysis of combined T2D and ND patients and analysis of T2D patients, marked as T2D patients).

LTA with ADP induction (%)	Prasugrel-treated patients	Clopidogrel-treated patients	Significance
Sample 1	45.9 ± 26.2%	59.2 ± 18.8%	*p* < 0.05
Sample 2	24.9 ± 17.7%	51.7 ± 22.8%	*p* < 0.001

LTA with ADP induction (%) T2D patients	Prasugrel-treated T2D patients	Clopidogrel-treated T2D patients	Significance

Sample 1	43.3 ± 20.6	63.5 ± 22.8	*p* < 0.05
Sample 2	22.9 ± 16.9	60.3 ± 26.6	*p* < 0.01

PRI of VASP-P analysis (%)	Prasugrel-treated patients	Clopidogrel-treated patients	Significance

Sample 1	52.7 ± 29.7%	68.9 ± 19.9	*p* < 0.05
Sample 2	26.3 ± 24.3%	51.8 ± 22.8%	*p* < 0.01

PRI of VASP-P analysis (%) T2D patients	Prasugrel-treated T2D patients	Clopidogrel-treated T2D patients	Significance

Sample 1	46.8 ± 33.5	78.3 ± 13.0	*p* < 0.05
Sample 2	18.7 ± 15.7	56.8 ± 18.7	*p* < 0.01

## Abbreviations

T2D: Type 2 diabetes
STEMI: ST elevation myocardial infarction
ADP: Adenosine diphosphate
VASP-P: Vasodilator-stimulated phosphoprotein phosphorylation
pPCI: Primary percutaneous coronary intervention
ND: Nondiabetic
ACS: Acute coronary syndrome
HTPR: High on-treatment platelet reactivity
HbA1C: Hemoglobin A1C
DCCT: Diabetes Control and Complications Trial
LTA: Light transmission aggregometry
PGE1: Prostaglandin E1
MFIc: Corrected mean fluorescence intensity
PRI: Platelet reactivity index
SD: Standard deviation
ECG: Electrocardiography
ED: Emergency department.
